# Factors associated with visual field or structure progression occurring first in a prospective study on patients with untreated open-angle glaucoma with normal intraocular pressure

**DOI:** 10.1038/s41433-023-02766-8

**Published:** 2023-10-05

**Authors:** Rei Sakata, Makoto Araie, Takeshi Yoshitomi, for lower normal pressure glaucoma study members in Japan Glaucoma Society, Takeshi Yoshitomi, Makoto Ishikawa, Haruki Abe, Takeo Fukuchi, Kazuhisa Sugiyama, Shinji Ohkubo, Koji Nitta, Makoto Araie, Atsuo Tomidokoro, Hiroyo Hirasawa, Keiji Yoshikawa, Aiko Iwase, Akira Negi, Yuko Yamada, Hidenobu Tanihara, Masaru Inatani, Toshihiro Inoue, Yuji Takihara, Saori Ohhira, Sachi Kojima, Chika Naitou, Motohiro Shirakashi, Tomomi Higashide, Chota Matsumoto, Sonoko Takada, Makoto Aihara, Hitomi Saito

**Affiliations:** 1 Department of Ophthalmology, Graduate of Medicine and Faculty of Medicine, The University of Tokyo, Tokyo, Japan; 2 Department of Ophthalmology, Kanto Central Hospital of the Mutual Aid Association of Public School Teachers, Tokyo, Japan; 3 Department of Orthoptics, Fukuoka International University of Health and Welfare, Fukuoka, Japan; 4 Department of Ophthalmology, Akita University Graduate School of Medicine, Akita, Japan; 5 Division of Ophthalmology and Visual Science, Graduate School of Medical and Dental Sciences, Niigata University, Niigata, Japan; 6 Department of Ophthalmology, Kanazawa University Graduate School of Medical Science, Ishikawa, Japan; 7 Department of Ophthalmology, Fukui-ken Saiseikai Hospital, Fukui, Japan; 8 Yoshikawa Eye Clinic, Tokyo, Japan; 9 Tajimi Iwase Eye Clinic, Gifu, Japan; 10 Division of Ophthalmology, Department of Surgery, Kobe University Graduate School of Medicine, Kobe, Japan; 11 Department of Ophthalmology, Faculty of Life Sciences, Kumamoto University, Kumamoto, Japan; 12 Kido Eye Clinic, Niigata, Japan; 13 Department of Ophthalmology, Kindai University Faculty of Medicine, Osaka-Sayama, Japan; 14 Takada Eye Clinic, Osaka-Sayama, Japan; 15 Present Address: Fukuoka International University of Health and Welfare, Fukuoka, Japan; 16 Present Address: Tohoku University, Sendai, Japan; 17 Present Address: Niigata University of Health and Welfare, Niigata, Japan; 18 Present Address: Ohkubo Eye Clinic, Ishikawa, Japan; 19 Present Address: Department of Ophthalmology, Kanagawa Dental University Yokoyama clinic, Kanagawa, Japan; 20 Present Address: Higashinakano Tomidokoro Eye Clinic, Tokyo, Japan; 21 Present Address: Toto Bunkyo Hospital, Tokyo, Japan; 22 Present Address: Biei City, Hokkaido, Japan; 23 Present Address: Department of Ophthalmology, Faculty of Medical Sciences, University of Fukui, Fukui, Japan

**Keywords:** Outcomes research, Risk factors

## Abstract

**Background/Objectives:**

To identify factors associated with disc/retina deterioration in stereo fundus photographs preceding that of the visual field (VF), as determined with a Humphrey Field Analyzer (HFA) (Structure First deterioration) and factors associated with the latter preceding the former (Field First deterioration) in open-angle glaucoma (OAG) with lower normal intraocular pressure (IOP).

**Subjects/Methods:**

Prospective cohort study. Ninety eyes of 90 patients with OAG and a baseline IOP < 15 mmHg participated in a 5-year prospective study without treatment. IOP measurements and HFA 24-2 Swedish Interactive Test Algorithm Standard tests were performed every 3 months, and fundus photographs were obtained every 6 months. VF deterioration was determined by Guided Progression Analysis and deterioration of disc/retina was determined on stereophotographs by an independent committee. A multivariable Cox proportional hazard model was used to identify factors associated with Structure First deterioration, and with Field First deterioration.

**Results:**

The average baseline age and mean deviation were 53.9 ± 9.8 years and −2.8 ± 2.8 dB, respectively. During the 5-year follow-up, the probability of Field First deterioration was 49% ± 6.6% (standard error) and that of Structure First deterioration was 33% ± 6.4% (*P* = 0.062, log-rank test). Disc hemorrhage (DH) prior to the event (*P* = 0.006) was associated with Structure First deterioration, and older age was associated with Field First deterioration (*P* = 0.040).

**Conclusions:**

In OAG eyes with lower normal IOP, DH was significantly associated with Structure First deterioration, and age was significantly associated with Field First deterioration.

## Introduction

Glaucoma is a leading cause of blindness; it was estimated to affect approximately 80 million people worldwide in 2020 [1]. Early detection and treatment of glaucoma remain the mainstays for preventing or delaying irreversible visual impairment [2]. It has been reported that 25–35% of retinal ganglion cells have already been lost by the time standard visual field (VF) testing detects abnormalities [3]. Optic nerve axonal damage in the lamina cribrosa and retinal ganglion cell death, which are reflected in structural changes in the disc and retina, are generally thought to precede VF defects detected using standard VF testing protocols [4]. Studies on experimental glaucomatous eyes in primates have shown that changes in the disc and lamina cribrosa occur prior to retinal nerve fibre layer (RNFL) changes and deterioration in visual function, as measured by multifocal electroretinography, with some time lag seen between the two events [5–8].

In humans, several longitudinal prospective studies with ≥ 5-year follow-up periods examined the relations between disc/retina changes and VF deterioration in patients with manifest glaucoma [9–13], suspected glaucoma [14], or ocular hypertension [9, 15]. Studies using confocal point scanning laser ophthalmoscopy (Heidelberg Retina Tomograph; Heidelberg Engineering GmbH, Heidelberg, Germany) and/or spectral-domain optical coherence tomography (SD-OCT) and a Humphrey Field Analyzer (HFA; Carl Zeiss Meditec, Jena, Germany) reported that progression was more likely to be detected first in the disc, and then in the VF, in manifest glaucoma patients after an estimated interval of 1–1.7 years [10–12]. Studies using stereo fundus photography and HFA reported similar findings in patients with suspected glaucoma [14] and ocular hypertension [15]. On the other hand, studies using stereo fundus photography and HFA in patients with manifest glaucoma reported that VF progression was more likely to precede disc/retina progression [9, 13].

Because missing disease progression would greatly reduce the efficacy of glaucoma treatment, identifying factors associated with Structure First or VF progression would be clinically useful for managing patients and may provide additional insights regarding the pathogenesis of glaucoma. To our knowledge, however, there have been no previous reports on this issue. We previously reported that the progression rate in patients with open-angle glaucoma (OAG) with a mean baseline intraocular pressure (IOP) of 12.3 mmHg for 5 years without treatment, and found that the rate of disease progression of 66% at 5 years was comparable to that reported in OAG patients with considerably higher IOP than in our cases [16]. In this cohort, we studied factors associated with documented disc/retina deterioration by stereo fundus photography preceding that of VF (Structure First deterioration), and factors associated with the latter preceding the former (Field First deterioration).

## Materials/subjects and methods

This multicentre prospective cohort study on the natural course of normal tension glaucoma (NTG) in apparently healthy Japanese patients who were followed up for 5 years without any medications was conducted as a part of the Japanese Lower Normal Pressure Glaucoma Study, supported by the Japanese Glaucoma Society[16]. All patients enrolled in the study were required to undergo evaluation of IOP and VF every 3 months, as well as optic disc stereophotography every 6 months for 5 years. During this period, no medical treatment was prescribed unless glaucoma progression (defined as VF deterioration and/or disc/retina deterioration) was confirmed. The inclusion and exclusion criteria are shown in Table 1. All patients provided written informed consent prior to enrollment. This study was performed in accordance with all relevant tenets of the Declaration of Helsinki, and the protocol was approved by the institutional review boards of the Office for Human Research Studies (OHRS), Graduate School of Medicine and Faculty of Medicine, The University of Tokyo (IRB number 1655) (registered ID: UMIN000001041).

**Table 1:** Study inclusion and exclusion criteria.

Inclusion criteria
1. No ocular pathologies other than open-angle glaucoma with a normal open angle
2. Aged 20–70 years on completion of the consent form
3. Glaucomatous disc/retinal changes corresponding to visual field defect according to the Anderson and Patella criteria^a^
4. Two consecutive reliable/reproducible visual field results were obtained at intervals of ≤ 3 months at the time of study entry
5. Mean deviation (MD) within 2 dB; MD > −15 dB in both eyes at commencement of observation
6. No application of intraocular pressure (IOP)-reducing agents for at least 6 months after such agents were discontinued before study entry
7. IOP < 15 mmHg without treatment on at least five of six consecutive IOP measurements during the 6 months before study entry
8. Absence of any intracranial disease that may affect the visual field
9. No clinically significant media opacity, including cataract

Exclusion criteria
1. Spherical equivalent < −9.0 or > +4.0 diopters
2. Best visual acuity < 0.8 (decimal)
3. History of intraocular surgery or laser treatment
4. History of uveitis, steroid use, trauma, ocular infection, or inflammatory disease
5. History of corneal disease, retinal disease, or cataract
6. History of diabetes mellitus or hypertension
7. Prescription of any drug that may affect IOP or ocular blood flow
8. Using antiplatelet and/or anticoagulant agents
9. Eyes with disc/retinal findings suggestive of any type of congenital anomaly, including strong myopic changes and tilted disc syndrome

^a^Anderson D. R., Patella V. M. Automated Static Perimetry. St. Louis, MO, USA: Mosby; 1999.

### Ophthalmoscopic examinations

Routine ophthalmic examinations were performed as follows. Slit-lamp and ophthalmoscopic examinations and IOP measurements were carried out every 3 months by Goldmann applanation tonometry (Haag-Streit Deutschland, Wedel, Germany) with the patient in a sitting position. VF tests were performed every 3 months during office hours using the Humphrey Field Analyzer Standard Swedish Interactive Threshold (SITA) Algorithm 24-2 program (HFA 24-2). Fundus stereophotographs were taken every 6 months after mydriasis of the eyes with a retinal camera (Nonmyd WX; Kowa, Tokyo, Japan) or auto fundus camera (AFC-330; NIDEK, Tokyo, Japan).

### Evaluation of visual field and disc/retina deterioration

The criteria for glaucoma progression in this study were as follows. VF deterioration was assessed using Guided Progression Analysis™ (GPA; Carl Zeiss Meditec). The average of the first two measurements of pattern deviation at each point of measurement constituted the baseline value. When significant progression (*P* < 0.05) was observed in three consecutive VF tests at three or more of the same points, the first of the three measurements was designated as the “deterioration time point” by the Visual Field Judging Committee (VFJC; C.M., A.I., and T. T). The following points were taken into consideration when determining the time point of VF deterioration. Although VF examination was performed every 3 months, the time point of VF deterioration was determined at an interval of 6 months (0, 6, 12 months, etc.), so that it coincided with the time point of disc/retina examination. Therefore, when the first of the three VF tests met the criteria of deterioration in an odd-numbered month, such as 15 months, the time point of VF deterioration was determined as 18 months. On the other hand, when the first of the three VF tests met the criteria of deterioration in an even-numbered month, such as 18 months, the time point of VF deterioration was determined as 18 months.

The disc/retina data were evaluated independently by the Optic Disc Reading Committee (ODRC; M.A., M.S., and T.H.) for consistency with the diagnosis of glaucoma. The committee agreed that the presented disc/peripapillary retina was definitely glaucomatous by referring to the VF only at entry, and confirmed that the structural change was consistent with VF defect on a hemifield basis. Definite and suspected hypoplastic optic disc, tilted disc syndrome, and ischemic disc were carefully excluded. The clinical data obtained during the follow-up period were completely masked from the members of the ODRC, who did not confer. Disc/retina deterioration was judged as follows. Two photographs (entry and follow-up photographs) were randomly marked A and B by a supporting division member (R.S.). The ODRC members, who were blinded to other findings, judged which of the two photographs showed more advanced glaucomatous change. The items checked included expansion of disc evacuation, focal marginal rim narrowing, and appearance or expansion of RNFL defects. The occurrence of DH was not a deterioration criterion. The ODRC members returned the final judgment to the manager. If the follow-up photographs were judged to be more advanced by all three ODRC members, the disc/retina in question was considered to satisfy the deterioration criteria (criterion 1). If two of the committee members agreed that the follow-up photograph showed a more advanced glaucomatous change, the final decision was arrived at by discussion, and if the follow-up photographs indicated more advanced disease, the disc/retina in question was considered to satisfy the deterioration criteria (criterion 2). Otherwise, the disc/retina was judged to be unchanged. A second judgment was made 6 months later. When the follow-up photographs were again judged to indicate deterioration according to criterion 1 or 2, the time when the disc/retina was first judged to have deteriorated was considered to be the time point of disc/retina deterioration.

### Evaluation of disc hemorrhage

The presence or absence of DH was checked carefully on photographs of the fundus with a view angle of 45° by three independent ODRC members, according to the same judgment criteria as outlined above, on a semi-annual basis. DH was considered to have occurred when it was present within one-third of the disc diameter from the optic disc border, within the optic disc, or within the retinal nerve fibre layer (RNFL), and was classified as splinter/flame-shaped hemorrhage not associated with optic disc edema, papillitis, diabetic retinopathy, central or branch retinal vein occlusion, or other retinal diseases. We considered DH that occurred within 6 months prior to enrolment or was found during the follow-up period (before the time point of disc/retina or VF deterioration). The presence or absence of DH in our study was based entirely on the determination from a stereo fundus photograph.

### Image analysis

Image analysis was performed using software (JGSTK Disc Analysis Soft; Topcon, Tokyo, Japan) [17, 18]. The vertical cup-to-disc ratio (v-C/D) and β-peripapillary atrophy (PPA) area/disc area ratio were calculated from stereo disc/retina and wide-angle photographs taken at baseline. Optic disc ovality was defined as the ratio between the longest diameter (LD) and shortest diameter (SD) of the optic disc (LD orthogonal to SD). The degree of optic disc torsion was measured between the LD and vertical meridian, which was identified as a vertical line (90°) emanating from a reference line connecting the fovea and center of the optic disc.

### Statistical analysis

The number of patients is set in the previous report [16]. When both eyes of one patient met the inclusion criteria, data from the eye with better mean deviation (MD) were used in the analysis. The primary outcomes were the probability of disc/retina deterioration Field First deterioration (Structure First deterioration) and the probability of VF deterioration Structure First deterioration (Field First deterioration), as well as the factors associated with these scenarios. Kaplan–Meier survival analysis was used to determine the time from baseline to Structure First or VF deterioration. A Cox proportional hazards model was used to identify risk factors for Structure First or VF deterioration. The ocular and systemic factors included as explanatory variables were age, spherical equivalent refraction, MD, pattern standard deviation (PSD), central corneal thickness (CCT), the v-C/D ratio, the β-parapapillary atrophy (PPA)/disc ratio, body mass index (BMI), systolic blood pressure (BP), diastolic BP, mean IOP during the follow-up period, IOP fluctuation (defined as the standard deviation of IOP during the follow-up period), the presence of DH before disc/retina or VF deterioration, optic disc ovality, and the degree of optic disc torsion. DH during the whole follow-up period was adopted in cases where both the disc/retina and VF were not judged to have deteriorated. The DH location and findings regarding disc/retina deterioration (i.e., disc evacuation expansion, focal marginal rim narrowing, and the appearance or expansion of the RNFL defect) were checked for consistency. The agreement of judging disc/retina deterioration among optic disc reading committees at 1 year was calculated from Fleiss Kappa.

All statistical analyses were performed with JMP 16.0 software (SAS Institute, Cary, NC, USA) and SPSS for Windows software (version 23.0; IBM Corp., Tokyo, Japan). All factors with a *P*-value < 0.15 in univariate regression analysis were included in multivariate analysis. In all analyses, *P* < 0.05 was taken to indicate statistical significance.

## Results

Ninety eyes of 90 patients with NTG were enrolled in the study. The baseline age, IOP and mean deviation (MD) were 53.9 ± 9.8 (standard deviation) years, 12.3 ± 1.2 mmHg and −2.8 ± 2.8 dB. The demographic and clinical data of the patients are shown in Table 2.

**Table 2:** Demographic and clinical characteristics of patients with NTG.

	Mean (SD)
Age (years)	53.9 (9.8)
Male/female	37/53
Right/left (eyes)	44/46
Spherical equivalent (diopters)	−3.5 (2.9)
Axial length (mm)^a^	25.0 (1.5)
Mean deviation (dB)	−2.8 (2.8)
Pattern standard deviation (dB)	5.1 (3.7)
IOP (baseline) (mmHg)	12.30 (1.15)
IOP (during follow-up) (mmHg)	12.35 (1.18)
Mean of the standard deviation of IOP during follow-up (mmHg)	1.19 (0.38)
Central corneal thickness (μm)	535.7 (26.0)
Body mass index	21.6 (3.0)
Systolic blood pressure (mmHg)	121 (16)
Diastolic blood pressure (mmHg)	76 (11)
Vertical cup/disc ratio	0.85 (0.053)
PPA-β/disc ratio	0.46 (0.46)
Optic disc ovality	1.26 (0.21)
Optic disc torsion (angle)	−10.7 (28.4)
Disc hemorrhage at entry (eyes)	9

*dB* decibel, *IOP* intraocular pressure, *NTG* normal tension glaucoma, *PPA* peripapillary atrophy, *SD* standard deviation.

^a^*n* = 78.

Of the 90 eyes examined, 32 showed Field First deterioration and 19 showed Structure First deterioration. Simultaneous VF and disc/retina deterioration occurred in one eye. Twenty-nine of thirty-two eyes showed further VF deterioration in areas where there was already VF deterioration at baseline, and three eyes showed VF deterioration in areas other than the original areas of VF damage. Thirteen of thirty-two eyes showed disc/retina deterioration following VF deterioration, with a mean interval of 18.5 ± 12.6 months (range: 6–42 months). On the other hand, 16 of 19 eyes with Structure First deterioration showed further deterioration in the area of original disc/retina changes, 2 eyes showed deterioration at a location of disc/retina other than that found at baseline, and 1 eye showed both. Six of nineteen eyes showed VF deterioration following Structure First deterioration, with a mean interval of 25.0 ± 11.6 months (range: 12–42 months) (*P* = 0.29 compared to Field First deterioration). There was consistency between the location of Structure First deterioration and that of subsequently detected VF deterioration, and vice versa, except in one case in which the Field First deterioration was followed by disc/retina deterioration at a different site. The judging agreement (Fleiss Kappa) of disc/retina deterioration was 0.65.

**Fig. 1:**
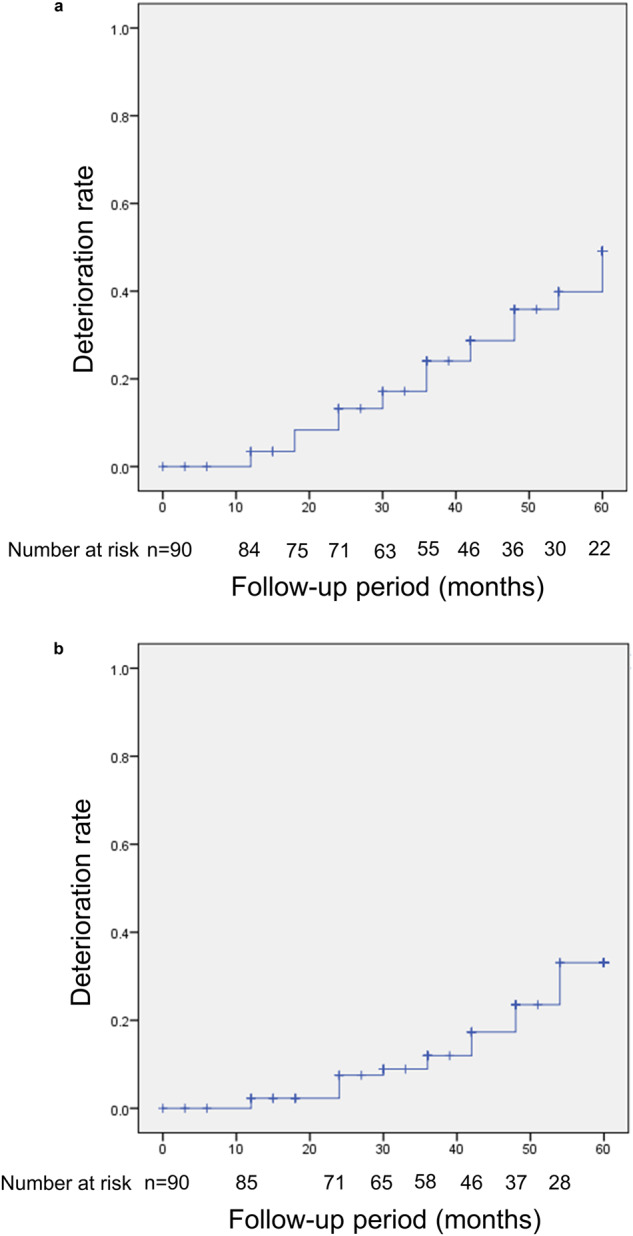
The deterioration rate of the visual field (a) and disc/retina (b). **a**. Kaplan–Meier survival analysis based on Field First deterioration. Vertical and horizontal axes indicate the deterioration rate (%) and time (months), respectively. **b** Kaplan–Meier survival analysis based on Structure First deterioration. Vertical and horizontal axes indicate the deterioration rate (%) and time (months), respectively.

The probability of Field First deterioration was 49% ± 6.6% (Fig. 1a), and that of Structure First deterioration was 33% ± 6.4% (Fig. 1b), at 5 years (*P* = 0.062, log-rank test). The multivariate Cox proportional hazard model showed that age was significantly associated with Field First deterioration (*P* = 0.040) (Table 3), and DH was significantly associated with Structure First deterioration (*P* = 0.006) (Table 4).

**Table 3:** Results of Cox’s proportional hazard model analysis of glaucoma progression (based on Field First deterioration).

Covariates	Univariate risk ratio (95%CI)	*P*-value	Multivariate risk ratio	*P*-value
Age^a^	1.04 (1.00–1.08)	0.050	1.04 (1.00–1.08)	0.040
Spherical equivalent^a^	1.06 (0.94–1.21)	0.328		
Mean deviation^a^	0.98 (0.87–1.14)	0.813		
Pattern standard deviation^a^	1.00 (0.89–1.10)	0.987		
Central corneal thickness^a^	1.00 (0.99–1.02)	0.555		
Vertical cup/disc ratio^a^	1.04 (0.98–1.12)	0.220		
PPA-β/disc ratio^a^	1.01 (0.93–1.09)	0.718		
Body mass index^a^	1.02 (0.90–1.14)	0.746		
Mean intraocular pressure^b^	1.01 (0.75–1.37)	0.926		
Intraocular pressure fluctuation^b^	1.08 (0.97–1.19)	0.143	1.09 (0.98–1.20)	0.113
Systolic blood pressure^a^	1.00 (0.98–1.03)	0.691		
Diastolic blood pressure^a^	1.00 (0.97–1.03)	0.909		
Optic disc ovality^a^	1.07 (0.89–1.24)	0.461		
Optic torsion degree^a^	1.00 (0.99–1.01)	0.759		
Disc hemorrhage^b^	1.52 (0.71–3.27)	0.293		

*CI* confidence interval, *PPA* peripapillary atrophy.

^a^at baseline, ^b^observed in the photographs during the follow-up.

**Table 4:** Results of Cox’s proportional hazard model analysis of glaucoma progression (based on Structure First deterioration).

Covariates	Univariate risk ratio (95%CI)	*P*-value	Multivariate risk ratio	*P*-value
Age^a^	1.02 (0.97–1.07)	0.417		
Spherical equivalent^a^	1.21 (1.02–1.46)	0.027	1.07 (0.86–1.35)	0.575
Mean deviation^a^	0.93 (0.82–1.11)	0.416		
Pattern standard deviation^a^	1.02 (0.89–1.15)	0.739		
Central corneal thickness^a^	0.99 (0.97–1.00)	0.081	0.98 (0.96–1.00)	0.119
Vertical cup/disc ratio^a^	0.98 (0.90–1.06)	0.563		
PPA-β/disc ratio^a^	0.87 (0.73–1.00)	0.054	0.98 (0.80–1.14)	0.788
Body mass index^a^	1.00 (0.84–1.17)	0.954		
Mean intraocular pressure^b^	0.65 (0.42–0.99)	0.043	0.81 (0.51–1.26)	0.360
Intraocular pressure fluctuation^b^	1.04 (0.90–1.20)	0.547		
Systolic blood pressure^a^	1.00 (0.98–1.03)	0.648		
Diastolic blood pressure^a^	1.01 (0.97–1.05)	0.723		
Optic disc ovality^a^	0.72 (0.50–0.98)	0.036	0.90 (0.56–1.39)	0.647
Optic torsion degree^a^	0.99 (0.97–1.00)	0.099	0.99 (0.98–1.01)	0.588
Disc hemorrhage^b^	5.06 (1.97–13.0)	<0.001	4.30 (1.51–12.3)	0.006

*CI* confidence interval, *PPA* peripapillary atrophy.

^a^at baseline, ^b^observed in the photographs during the follow-up.

## Discussion

The main goal of this study was to identify factors associated with disc/retina deterioration in stereo fundus photographs preceding that of VF as determined with HFA 24-2 (Structure First deterioration) and factors associated with the latter preceding the former (Field First deterioration) in untreated OAG patients with lower normal IOP of 12.3 mmHg.

The probability of Field First deterioration was 49% at 5 years, with older age being a significant risk factor, and that of Structure First was 33%, with DH prior to the event being a significant risk factor. Despite the higher probability of Field First deterioration (*P* = 0.062), DH was not a significant risk factor even in preliminary univariate analysis. As the statistical power of the analysis to detect risk factors in this case should not have been inferior to that in the case of Structure First deterioration, the contribution of DH as the risk was thought to be more evident in the case of Structure First deterioration.

Previous reports where VF and disc/retina deterioration were considered separately during follow-up all appeared to include treated cases [9–13, 19]. However, it should be noted that IOP-lowering therapy may modify not only the course of glaucoma progression but also the frequency of DH in eyes [20]. In this respect, the present cohort was unique in that it was free of IOP-lowering therapy before further deterioration of disc/retina or VF was confirmed. The only previous report including untreated patients was the Ocular Hypertension Treatment Study (OHTS) [15], where progression was judged by stereo disc photography and HFA-measured VF, as in the present study. Of the 819 eyes in the no-treatment group (mean IOP of 24.9 mmHg), 89 eyes developed glaucoma, of which 51 (57%) had disc/retina deterioration first, 29 (33%) had VF deterioration first, and the remaining 9 had concurrent deterioration of disc/retina and VF. The higher percentage of Structure First deterioration in OHTS (57% vs. 33%) was in contrast to the lower percentage of Structure First deterioration in the present cohort, for which the mean IOP was 12.3 mmHg (33% vs. 49%), suggesting a difference between the two populations, such as vertical C/D ratio at the initial examination (OHTS: 0.39 versus ours: 0.85) or in the contribution of IOP-dependent damage processes to the relation between subsequent disc/retina and VF deterioration. Another possibility is that the rate of prior VF or disc/retina deterioration may vary depending on whether VF loss or disc/retina glaucomatous changes are apparent or not. Our study included all diagnosed glaucoma patients, already exhibiting visual field abnormalities. This differs from the OH patients who had not yet developed glaucomatous optic neuropathy, thereby leading to a different rate of progression.

DH is seen more frequently in glaucomatous than healthy eyes, with the highest rate of occurrence in NTG seen in the early to moderate stages [21, 22]. However, the mechanism by which DH emerges is complex and not yet fully understood. In addition to the possible involvement of IOP-dependent factors in the development of DH [20, 23], it has been suggested that vascular abnormalities may also play a role [18]. For example, the frequency of DH was found to be higher in patients with DM [24], hypertension [25], and lower systolic BP [26]. Moreover, associations with aspirin use [24] and antiplatelet medications [27] were also reported, suggesting that abnormal microcirculatory status may result in a predisposition to microvasculature disruption of the tissue around the optic disc [28]. As the subjects in the present study were free of these circulatory abnormalities, it seems unlikely that DH itself first occurred, triggering the subsequent events. It has been reported that DH often appears at or near sites of glaucomatous disc/retina changes (rim notch [29, 30], RNFL defect border [31, 32], and site of PPA [33]), suggesting that DH is closely related to structural damage of the disc/retina. It has been also reported that RNFL loss [34] and VF deterioration [35] progressed before the occurrence of DH. The results presented here showing that DH was associated with Structure First deterioration but not with Field First deterioration were not incompatible with these previous studies. It is not clear from the present results which of the neuronal or non-neuronal components around the disc/retina was more closely related to DH. The results of the present study also suggested that there may be two different types of glaucoma progression even in OAG eyes with lower normal pressure, on the assumption of less involvement of IOP-dependent glaucomatous insults and confounding effects of microcirculatory abnormalities: one where DH was associated with subsequent disc/retina deterioration occurring before functional damage, and another where some as yet unknown aging-associated abnormalities were associated with functional damage detected before disc/retina deterioration. It is interesting to note that glaucoma eyes with documented disc/retina deterioration during follow-up reportedly showed more rapid VF deterioration than those with documented deterioration of VF but not disc/retina during follow-up [19].

This study had several limitations. If VF progression occurred in an odd-numbered month, the decision was made 3 months later due to an adjustment made to the testing intervals for the VF (every 3 months) and disc/retina (every 6 months). This may have resulted in a delay of VF deterioration assessment, thus leading to an underestimation of the probability of Field First deterioration. In the present study, however, no eyes with Structure First deterioration showed subsequent VF deterioration within 6 months of disc/retina deterioration. Second, the 5-year follow-up of the present cohort may have been too short relative to the duration of OAG. It should be noted that our conclusions were based on the results of about 58% of all subjects who showed disease progression during the 5-year follow-up period. Third, slight changes in the disc/retina may have been detected more sensitively using SD-OCT. However, SD-OCT was not in wide clinical use at the start of the study. If SD-OCT had been utilized, the proportion of Structure First deterioration may have increased, and the results would be different this time, and also for its association with DH. Further, if the SD-OCT-derived Bruch’s membrane opening-minimum rim width, which mainly reflects neuronal components [36], had been measured, more information on the pathogenesis of DH may have been obtained. Fourth, a study of NTG eyes reported that the average duration of DH was 10.6 weeks [37]. Therefore, the fundus photographs taken at 3-month (12-week) intervals could miss the presence of DH. In the present study, the rate of missing DH was thought to be even higher because fundus photographs were taken at 6-month (24-week) intervals. However, even if we had increased the frequency of fundus examination and were able to detect DH more frequently, this effect would also have similarly affected the results of disc/retina deterioration and VF deterioration, and we do not think it would greatly alter the observation that DH was more likely to be associated with Structure First deterioration than Field First deterioration. Moreover, given the known correlation between DH and structural deterioration, one might think that the presence of DH might have caused some bias in the determination of disc/retinal deterioration. However, since the committee was not given any information regarding the presence or absence of DH in the past, it was difficult that a bias could have arisen. Finally, the present results were obtained from OAG patients with IOP consistently below the average normal IOP (15 mmHg) without treatment, rather than OAG patients with elevated IOP.

In untreated OAG patients with a mean baseline IOP of 12.3 mmHg, DH was a significant risk factor for Structure First deterioration, which had a probability of 33% at 5 years. Moreover, older age was a significant risk factor for Field First deterioration, with a probability of 49% at 5 years. DH as a risk factor was thought to be more important in the case of Structure First deterioration. Although both VF and disc/retina must be vigilantly monitored to accurately track the disease’s progression, our results would suggest that when DH is observed in OAG patients with lower normal IOP, more attention should be paid to disc/retina findings for early detection of progression, while in older patients without DH attention should be paid to VF.

## Summary

### What was known before

- Although there have been several longitudinal reports examining which deteriorates first between the function (visual field) and the structure (disc/retina) in glaucomatous eyes, there has been no similar research in patients with only normal-tension glaucoma (NTG).
- In addition, these previous reports included medical treatments with eye drops, thus, the impact of treatment on the glaucoma progression could not be ignored. Further, factors associated with preceding deterioration have not been studied.

### What this study adds

- This 5-year prospective study investigated the preceding deterioration between the visual field (Field) and disc/retina (Structure) in eyes with untreated NTG and elucidated its related factors.
- A higher ratio of Field First deterioration was found than in Structure First deterioration in eyes with untreated NTG. Disc hemorrhage was significantly associated with Structure First deterioration and age was significantly associated with Field First deterioration.
- Given these results, although VF is more likely to progress in eyes with NTG, we should pay more attention to disc/retina findings than VF for early detection of progression when DH is observed in real clinical practice.

### Supplementary information

eye-reporting-checklist

ICMJE DISCLOSURE FORM

ICMJE DISCLOSURE FORM

ICMJE DISCLOSURE FORM

## Data Availability

The datasets generated during and/or analyzed during the current study are not publicly available due [under creating a relevant report].

## References

[1] Tham YC, Li X, Wong TY, Quigley HA, Aung T, Cheng CY (2014). Global prevalence of glaucoma and projections of glaucoma burden through 2040: a systematic review and meta-analysis. Ophthalmology.

[2] Weinreb RN, Aung T, Medeiros FA (2014). The pathophysiology and treatment of glaucoma: a review. JAMA.

[3] Kerrigan-Baumrind LA, Quigley HA, Pease ME, Kerrigan DF, Mitchell RS (2000). Number of ganglion cells in glaucoma eyes compared with threshold visual field tests in the same persons. Invest Ophthalmol Vis Sci.

[4] Weinreb RN, Friedman DS, Fechtner RD, Cioffi GA, Coleman AL, Girkin CA (2004). Risk assessment in the management of patients with ocular hypertension. Am J Ophthalmol.

[5] Strouthidis NG, Fortune B, Yang H, Sigal IA, Burgoyne CF (2011). Longitudinal change detected by spectral domain optical coherence tomography in the optic nerve head and peripapillary retina in experimental glaucoma. Invest Ophthalmol Vis Sci.

[6] Fortune B, Burgoyne CF, Cull GA, Reynaud J, Wang L (2012). Structural and functional abnormalities of retinal ganglion cells measured in vivo at the onset of optic nerve head surface change in experimental glaucoma. Invest Ophthalmol Vis Sci.

[7] Fortune B, Reynaud J, Wang L, Burgoyne CF (2013). Does optic nerve head surface topography change prior to loss of retinal nerve fiber layer thickness: a test of the site of injury hypothesis in experimental glaucoma. PLoS One.

[8] He L, Yang H, Gardiner SK, Williams G, Hardin C, Strouthidis NG (2014). Longitudinal detection of optic nerve head changes by spectral domain optical coherence tomography in early experimental glaucoma. Invest Ophthalmol Vis Sci.

[9] Miglior S, Brigatti L, Lonati C, Rossetti L, Pierrottet C, Orzalesi N (1996). Correlation between the progression of optic disc and visual field changes in glaucoma. Curr Eye Res.

[10] Chauhan BC, McCormick TA, Nicolela MT, LeBlanc RP (2001). Optic disc and visual field changes in a prospective longitudinal study of patients with glaucoma: comparison of scanning laser tomography with conventional perimetry and optic disc photography. Arch Ophthalmol.

[11] Chauhan BC, Nicolela MT, Artes PH (2009). Incidence and rates of visual field progression after longitudinally measured optic disc change in glaucoma. Ophthalmology.

[12] Xu G, Weinreb RN, Leung CK (2014). Optic nerve head deformation in glaucoma: the temporal relationship between optic nerve head surface depression and retinal nerve fiber layer thinning. Ophthalmology.

[13] Öhnell H, Heijl A, Brenner L, Anderson H, Bengtsson B (2016). Structural and Functional Progression in the Early Manifest Glaucoma Trial. Ophthalmology.

[14] Medeiros FA, Alencar LM, Zangwill LM, Bowd C, Sample PA, Weinreb RN (2009). Prediction of functional loss in glaucoma from progressive optic disc damage. Arch Ophthalmol.

[15] Kass MA, Heuer DK, Higginbotham EJ, Johnson CA, Keltner JL, Miller JP (2002). The Ocular Hypertension Treatment Study: a randomized trial determines that topical ocular hypotensive medication delays or prevents the onset of primary open-angle glaucoma. Arch Ophthalmol.

[16] Sakata R, Yoshitomi T, Iwase A, Matsumoto C, Higashide T, Shirakashi M (2019). Factors Associated with Progression of Japanese Open-Angle Glaucoma with Lower Normal Intraocular Pressure. Ophthalmology.

[17] Saito H, Tsutsumi T, Iwase A, Tomidokoro A, Araie M (2010). Correlation of disc morphology quantified on stereophotographs to results by Heidelberg Retina Tomograph II, GDx variable corneal compensation, and visual field tests. Ophthalmology.

[18] Iwase A, Sawaguchi S, Sakai H, Tanaka K, Tsutsumi T, Araie M (2017). Optic disc, rim and peripapillary chorioretinal atrophy in normal Japanese eyes: the Kumejima Study. Jpn J Ophthalmol.

[19] De Moraes CG, Liebmann JM, Park SC, Teng CC, Nemiroff J, Tello C (2013). Optic disc progression and rates of visual field change in treated glaucoma. Acta Ophthalmol.

[20] Hendrickx KH, van den Enden A, Rasker MT, Hoyng PF (1994). Cumulative incidence of patients with disc hemorrhages in glaucoma and the effect of therapy. Ophthalmology.

[21] Jonas JB, Xu L (1994). Optic disk hemorrhages in glaucoma. Am J Ophthalmol.

[22] Yamamoto T (2019). The impact of disc hemorrhage studies on our understanding of glaucoma: a systematic review 50 years after the rediscovery of disc hemorrhage. Jpn J Ophthalmol.

[23] Miyake T, Sawada A, Yamamoto T, Miyake K, Sugiyama K, Kitazawa Y (2006). Incidence of disc hemorrhages in open-angle glaucoma before and after trabeculectomy. J Glaucoma.

[24] Soares AS, Artes PH, Andreou P, Leblanc RP, Chauhan BC, Nicolela MT (2004). Factors associated with optic disc hemorrhages in glaucoma. Ophthalmology.

[25] Kim YD, Han SB, Park KH, Kim SH, Kim SJ, Seong M (2010). Risk factors associated with optic disc haemorrhage in patients with normal tension glaucoma. Eye (Lond).

[26] Furlanetto RL, De Moraes CG, Teng CC, Liebmann JM, Greenfield DS, Gardiner SK (2014). Risk factors for optic disc hemorrhage in the low-pressure glaucoma treatment study. Am J Ophthalmol.

[27] Grødum K, Heijl A, Bengtsson B (2002). Optic disc hemorrhages and generalized vascular disease. J Glaucoma.

[28] Quigley HA, Addicks EM, Green WR, Maumenee AE (1981). Optic nerve damage in human glaucoma. II. The site of injury and susceptibility to damage. Arch Ophthalmol.

[29] Law SK, Choe R, Caprioli J (2001). Optic disk characteristics before the occurrence of disk hemorrhage in glaucoma patients. Am J Ophthalmol.

[30] Airaksinen PJ, Mustonen E, Alanko HI (1981). Optic disc hemorrhages. Analysis of stereophotographs and clinical data of 112 patients. Arch Ophthalmol.

[31] Sugiyama K, Uchida H, Tomita G, Sato Y, Iwase A, Kitazawa Y (1999). Localized wedge-shaped defects of retinal nerve fiber layer and disc hemorrhage in glaucoma. Ophthalmology.

[32] Nitta K, Sugiyama K, Higashide T, Ohkubo S, Tanahashi T, Kitazawa Y (2011). Does the enlargement of retinal nerve fiber layer defects relate to disc hemorrhage or progressive visual field loss in normal-tension glaucoma?. J Glaucoma.

[33] Jonas JB, Martus P, Budde WM, Hayler J (2002). Morphologic predictive factors for development of optic disc hemorrhages in glaucoma. Invest Ophthalmol Vis Sci.

[34] Jeoung JW, Park KH, Kim JM, Kang SH, Kang JH, Kim TW (2008). Optic disc hemorrhage may be associated with retinal nerve fiber loss in otherwise normal eyes. Ophthalmology.

[35] De Moraes CG, Prata TS, Liebmann CA, Tello C, Ritch R, Liebmann JM (2009). Spatially consistent, localized visual field loss before and after disc hemorrhage. Invest Ophthalmol Vis Sci.

[36] Chauhan BC, O’Leary N, AlMobarak FA, Reis ASC, Yang H, Sharpe GP (2013). Enhanced detection of open-angle glaucoma with an anatomically accurate optical coherence tomography-derived neuroretinal rim parameter. Ophthalmology.

[37] Kitazawa Y, Shirato S, Yamamoto T (1986). Optic disc hemorrhage in low-tension glaucoma. Ophthalmology.

